# Physiological Effects of TolC‐Dependent Multidrug Efflux Pumps in *Escherichia coli*: Impact on Motility and Growth Under Stress Conditions

**DOI:** 10.1002/mbo3.70006

**Published:** 2024-11-11

**Authors:** Amanda M. Di Maso, Cristian Ruiz

**Affiliations:** ^1^ Department of Biology California State University Northridge Northridge California USA

**Keywords:** AcrAB‐TolC, EmrAB‐TolC, EmrKY‐TolC, MacAB‐TolC, MdtABC‐TolC, MdtEF‐TolC

## Abstract

Enterobacteriaceae possess eight TolC‐dependent multidrug efflux pumps: AcrAB‐TolC, AcrAD‐TolC, AcrEF‐TolC, MdtEF‐TolC, MdtABC‐TolC, EmrAB‐TolC, EmrYK‐TolC, and MacAB‐TolC, which efflux bile salts, antibiotics, metabolites, or other compounds. However, our understanding of their physiological roles remains limited, especially for less‐studied pumps like EmrYK‐TolC. In this study, we tested the effects on swimming motility and growth under stress conditions of *Escherichia coli* mutants individually deleted for each inner‐membrane transporter component of all eight TolC‐dependent pumps, a mutant deleted for the AcrB‐accessory protein AcrZ, and a mutant simultaneously deleted for all eight pumps (Δ*tolC*). We found that all mutants tested, except the Δ*emrY* and Δ*acrZ* mutants, displayed increased swimming motility. Additionally, the loss of each individual TolC‐dependent pump or AcrZ did not reduce growth and sometimes even enhanced it compared to the parental strain under various growth conditions: temperature (LB at 25, 30, 37, and 42°C), pH (LB at pH 6.0, 7.4, and 9.0; and LB buffered to pH 6.0, 7.4, and 8.25), LB with limited air exchange, and nutritional stress (M9‐glucose or M9‐glycerol). In contrast, the Δ*tolC* mutant grew significantly slower than the parental strain under all conditions tested except in LB‐TRIS pH 7.4 and LB with limited air exchange. Overall, these findings indicate that while individual TolC‐dependent pumps are generally dispensable for growth under many stress conditions in the absence of antimicrobials, possibly due to their partially overlapping substrate profiles, TolC‐dependent efflux is required for maximal growth under most conditions.

## Graphical abstract

The eight TolC‐dependent multidrug efflux pumps of *Escherichia coli* confer resistance to antibiotics. We report that while individual TolC‐dependent pumps are dispensable for growth under many stress conditions in the absence of antimicrobials, possibly due to their partially overlapping substrate profiles, TolC‐dependent efflux is required for maximal growth under most conditions.

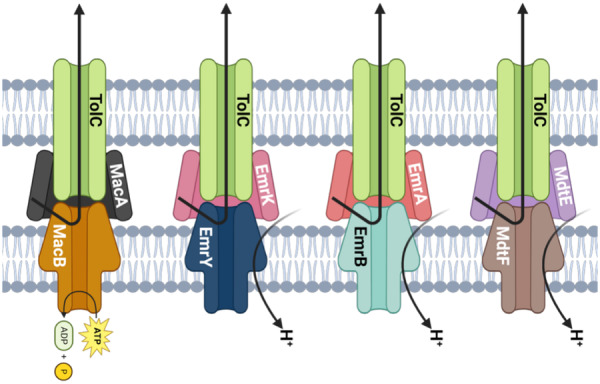

## 1 Introduction

**Figure 1:**
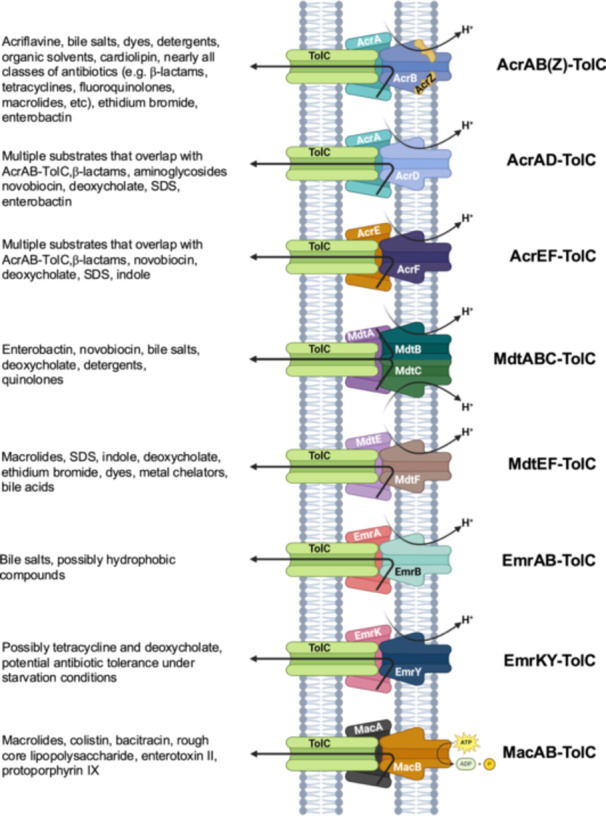
Schematics of the eight TolC‐dependent efflux pumps of *E. coli*, their mechanism of action, and their major substrates. Substrate profile is summarized mostly from references (Li, Plésiat, and Nikaido 2015; Teelucksingh et al. 2022).

Efflux pumps are well‐known to contribute to the growing antibiotic resistance threat by providing an effective mechanism to expel antimicrobials from bacterial cells, thereby conferring antibiotic resistance (Nanjan and Bose 2024; Du et al. 2018; Li, Plésiat, and Nikaido 2015). AcrAB‐TolC is considered the primary multidrug efflux (MDR) pump under most growth conditions in *Escherichia coli* as well as other species in the *Enterobacteriaceae* family (Li, Plésiat, and Nikaido 2015; Anes et al. 2015; Tal and Schuldiner 2009). This pump effluxes most classes of antibiotics and other toxic antimicrobial compounds such as bile salts and detergents (Li, Plésiat, and Nikaido 2015; Sulavik et al. 2001; Nikaido and Pagès 2012; Teelucksingh et al. 2022; Nishino and Yamaguchi 2001). In *E. coli*, TolC forms the outer membrane channel and creates large protein complexes that span the envelope with eight known MDR pumps: AcrAB, AcrAD, AcrEF, EmrAB, EmrKY, MacAB, MdtABC, and MdtEF (Nanjan and Bose 2024; Du et al. 2018; Li, Plésiat, and Nikaido 2015) (Figure 1). The efflux profiles of these eight pumps have been studied mainly regarding their ability to export antimicrobial compounds, and have been found to overlap considerably for several of them (Li, Plésiat, and Nikaido 2015; Sulavik et al. 2001; Nikaido and Pagès 2012; Teelucksingh et al. 2022; Nishino and Yamaguchi 2001). In other cases, such as EmrKY‐TolC, their substrate profile is not well understood yet (Pasqua et al. 2019). Moreover, the role of pumps such as AcrEF‐TolC and EmrKY‐TolC on antibiotic resistance is mostly apparent only when overexpressed in an AcrAB‐TolC‐free background (Li, Plésiat, and Nikaido 2015; Sulavik et al. 2001; Nikaido and Pagès 2012; Teelucksingh et al. 2022; Nishino and Yamaguchi 2001; Nishino et al. 2021; Tanabe et al. 1997; Lau and Zgurskaya 2005; Zhang et al. 2018).

Members of the resistance‐nodulation‐division (RND) family of pumps (AcrAB, AcrAD, AcrEF, MdtABC, and MdtEF) as well as the members of the major facilitator superfamily (MFS) of pumps (EmrAB, EmrKY) are antiporters that efflux substrates using proton motive force (PMF)‐powered peristaltic motion (Nanjan and Bose 2024; Du et al. 2018; Li, Plésiat, and Nikaido 2015). Of the TolC‐dependent MDR pumps, only MacAB, a member of the ATP‐binding cassette (ABC) family (Fitzpatrick et al. 2017) uses ATP hydrolysis to power its substrate efflux (Nanjan and Bose 2024; Du et al. 2018; Li, Plésiat, and Nikaido 2015). The inner membrane component of all these pumps captures the pump substrates and conveys them to the TolC channel protein for extracellular export (Nanjan and Bose 2024; Du et al. 2018; Li, Plésiat, and Nikaido 2015). In AcrAB‐TolC, the AcrA component serves as a periplasmic adaptor protein that connects TolC to the inner membrane transporter component, AcrB (Nanjan and Bose 2024; Du et al. 2018; Li, Plésiat, and Nikaido 2015; Du et al. 2014). This structure is repeated in the other TolC‐dependent pumps, except for MdtABC‐TolC (Kim, Nagore, and Nikaido 2010). In this pump, two inner membrane components, MdtB and MdtC, are required alongside the MdtA periplasmic adaptor and TolC for pump functionality (Nanjan and Bose 2024; Du et al. 2018; Li, Plésiat, and Nikaido 2015; Kim, Nagore, and Nikaido 2010). In addition, the AcrB component of the AcrAB‐TolC is known to form a complex with the small protein AcrZ, which is highly conserved among the Enterobacteriaceae (Du et al. 2014; Hobbs et al. 2012). *acrZ* is transcriptionally activated by MarA, SoxS, and Rob (Hobbs et al. 2012), which are also known to activate transcription of *acrAB* and *tolC* (Rosenberg et al. 2003; Martin and Rosner 2011; Zhang, Rosner, and Martin 2008; Rosner and Martin 2009; Ruiz and Levy 2014, 2010). AcrZ is thought to modulate the binding of AcrB to a subset of its substrates (Hobbs et al. 2012), as well as its interaction with cardiolipin in the inner membrane (Du et al. 2020).

Strong evidence continues to emerge that TolC‐dependent MDR pumps have important physiological roles beyond effluxing bile salts and other antimicrobials, including roles in metabolism, gene expression, cell signaling, stress responses, biofilm formation, and motility (Ruiz and Levy 2014; Cauilan et al. 2019; Harmon and Ruiz 2022; Maldonado et al. 2023; Shirshikova et al. 2021; Webber et al. 2009). For example, we have found that an *acrB*‐deleted (Δ*acrB*) *E. coli* mutant is hypermotile (Ruiz and Levy 2014; Maldonado et al. 2023). This phenotype seems to be mostly mediated by the inactivation of the *acrAB* transcriptional repressor AcrR, which is also a direct repressor of the *flhDC* master regulator of motility, by cellular metabolites that accumulate in the Δ*acrB* mutant (Ruiz and Levy 2014; Maldonado et al. 2023). When swimming motility was studied in *Salmonella enterica*, a mutant lacking *acrB* (*acrB::aph*) was found to be hypomotile (Webber et al. 2009), whereas a point *acrB* mutant (D408A) impaired for efflux was hypermotile (Wang‐Kan et al. 2017). In *Serratia marcescens*, the Δ*macAB* mutant has been found to display decreased motility, biofilm formation, and survival in the presence of oxygen peroxide (Shirshikova et al. 2021). Decreased biofilm formation has also been found in *E. coli* mutants lacking *acrD*, *acrE*, *mdtE*, or *emrK* genes (Matsumura et al. 2011).

In addition, we have found broad changes in gene expression in the *E. coli* Δ*acrB* mutant (Ruiz and Levy 2014), and in the intracellular and extracellular metabolic profiles of the Δ*acrB* and Δ*tolC E. coli* mutants compared to the parental strain (Cauilan et al. 2019). AcrAB‐TolC, as well as AcrAD‐TolC and MdtABC‐TolC, have also been found to be required for the export of the siderophore enterobactin (Horiyama and Nishino 2014). The metabolic intermediate sodium malonate has been shown to inhibit the AcrAB‐TolC pump (Cauilan and Ruiz 2022). AcrEF‐TolC has been suggested to play a minor role in exporting indole (Pinero‐Fernandez et al. 2011). MdtEF‐TolC confers a fitness advantage when *E. coli* is exposed to anaerobic or acid conditions (Zhang et al. 2011; Deng et al. 2013; Schaffner et al. 2021) and contributes to survival in macrophages (Fanelli et al. 2023). Expression of *emrKY* is upregulated under acidic stress conditions and inside macrophages (Weiss and Schaible 2015), and has been suggested to export toxic byproducts of DNA damage from exposure to UV radiation or reactive oxygen species in the macrophage (Pasqua et al. 2019). MacAB‐TolC is known to efflux virulence factors such as rough core lipopolysaccharide (Lu and Zgurskaya 2013) and heat‐stable enterotoxin II (Yamanaka et al. 2008), as well as contributes to iron homeostasis by exporting the heme precursor protoporphyrin IX (Turlin et al. 2014).

Overall, all these findings indicate an important physiological role of the TolC‐dependent MDR efflux pumps of *E. coli* and other Enterobacteriaceae, particularly in metabolism, mitigation of pH and oxidative stress, during anaerobic growth, and survival in the macrophage. However, our understanding of the physiological roles of these pumps remains limited. Moreover, the individual contributions of each of these eight pumps to different physiological functions and growth under different stress conditions are still poorly understood, especially because they have often been studied in different species, strains or growth conditions; or in the case of pumps such EmrKY‐TolC, barely studied at all. To contribute to filling this knowledge gap, here we report the first comprehensive characterization of *E. coli* mutants individually deleted for each of the eight TolC‐dependent pumps, and the Δ*acrZ* and Δ*tolC* mutants, all in the same genetic background and studied side‐by‐side under the same growth conditions. We have examined the effect of these mutants on swimming motility and growth under temperature, pH, limited air exchange, and nutritional stress. Our findings suggest a dynamic role of the TolC‐dependent pumps of *E. coli* during growth under different conditions that allows other TolC‐dependent pumps to compensate for the absence of any individual pump, whereas simultaneous inactivation of all of them significantly diminishes growth under nearly all conditions tested.

## 2 Materials and Methods

### 2.1 Bacterial Strains and Cultures

The *Escherichia coli* parental strain and TolC‐dependent efflux mutants used in this study are listed in Table 1. All strains were verified by PCR using the gene‐specific primers listed in Table 2. PCR reactions were performed using the DreamTaq polymerase from Thermo Fisher Scientific (Waltham, MA, USA) as recommended by the manufacturer, followed by visualization in 1% agarose gels stained with 0.5 µg/mL of ethidium bromide.

**Table 1:** *Escherichia coli* strains used in this study.

Strain	Description	Source/Reference
BW25113	(Parental) F^–^ λ^–^ Δ*(araD–araB)567* Δ*lacZ4787*(::*rrnB‐3*) *rph‐1* Δ*(rhaD–rhaB)568 hsdR514*	CGSC, Keio collection Baba et al. (2006)
JW0451	BW25113 Δ*acrB*::*kan*	Baba et al. (2006)
JW2454	BW25113 Δ*acrD*::*kan*	Baba et al. (2006)
JW3234	BW25113 Δ*acrF*::*kan*	Baba et al. (2006)
JW2661	BW25113 Δ*emrB*::*kan*	Baba et al. (2006)
JW2364	BW25113 Δ*emrY*::*kan*	Baba et al. (2006)
JW0863	BW25113 Δ*macB*::*kan*	Baba et al. (2006)
JW2060	BW25113 Δ*mdtB*::*kan*	Baba et al. (2006)
JW2061	BW25113 Δ*mdtC*::*kan*	Baba et al. (2006)
JW3482	BW25113 Δ*mdtF*::*kan*	Baba et al. (2006)
JW5503	BW25113 Δ*tolC*::*kan*	Baba et al. (2006)
JW5102	BW25113 Δ*acrZ*::*kan*	Baba et al. (2006)

**Table 2:** Primers used to verify the strains used in this study.

Gene	Primer name – 5′ to 3′ sequence	*Tm* (°C)	Reference
*acrB*	acrBF – TAAACAGGAGCCGTTAAGAC acrBRR – CGCGGCCTTAGTGATTACAC	54.3 57.7	Ruiz and Levy (2014)
*acrD*	acrDgF – CCTACAACGATACGCAGAAA acrDgR – CGCTGAGCAGGTTCTTAAT	52.3 52.5	This study
*acrF*	acrFgF – GCATCGAAGTAAGGTAATCTG acrFgR – ATGGGTTTCACTGGAAATAA	50.8 49.3	This study
*mdtB*	mdtBgF – AAGTGGAAGTGGTGGAAG mdtBgR – ATGGCAACCGACAGTAAA	51.9 51.6	This study
*mdtC*	mdtCgF – GGTGCTGACGCTGTTTAC mdtCgR – AAGCCGAAAGCCACAATC	54.0 53.5	This study
*mdtF*	mdtFgF – ACGAGCAATTTCCTCCAG mdtFgR – GACGGTTAGCTGGTTGTT	52.3 52.5	This study
*emrB*	emrBgF – AGCTAACGCTGGCTAATC emrBgR – GCAGGAACTGCACATCTA	52.1 52.0	This study
*emrY*	emrYgF – TCGATACCAGTCCGATAGA emrYgR – CAGCATCGCAATCCTTTC	51.5 51.7	This study
*macB*	macBgF – CACGTAACGATACCGATGT macBgR – TGTGTACATCCTAAAGGCAA	51.9 51.3	This study
*tolC*	tolCF – TGCTTCACCACAAGGAATGC tolCR – CCGAAGCCCCGTCGTCGTCA	57.7 57.7	Ruiz and Levy (2014)
*acrZ*	acrZgF – CTGTGCTTAGCGGTTAGA acrZgR – GCTAACCTTTGTGAGGTAGA	51.7 51.6	This study

*E. coli* strains were routinely grown in lysogeny broth (LB; 5 g/L yeast extract, 10 g/L tryptone, 10 g/L NaCl) or LB agar (LB with 15 g/L agar). Mutant strains were maintained on LB agar plates containing 50 µg/mL kanamycin (LB‐kan).

### 2.2 Motility Experiments

**Figure 2:**
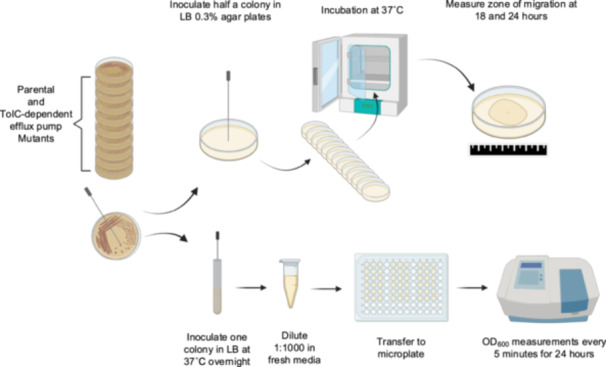
Schematics of the experimental approach used for motility and growth experiments under different temperatures, pH, limited air exchange and nutritional stress conditions. Full details are provided in the materials and methods section.

Swimming motility was measured by inoculating strains onto LB motility plates with a low (0.3% w/v) agar content as described by Ruiz and Levy (Ruiz and Levy 2014), using large (150 mm diameter) plates. Half of a single colony for each strain was inoculated onto the center of a single LB motility plate using a sterile toothpick; the other half of the colony was used to inoculate another plate for a technical replicate. Plates were then incubated for up to 24 h at 37°C in a plastic zipper storage bag to maintain humidity. At 18‐ and 24‐h postinoculation, the diameter of the zone of migration was measured in perpendicular axes and then averaged for the final diameter of migration (Figure 2). Each strain was tested using four to eight biological replicates each one consisting of two technical replicates.

### 2.3 Growth Curve Experiments in LB at 37°C

To determine whether the results observed in the motility assays were affected by differences in growth between strains, cultures of all strains were grown overnight in LB at 37°C with 200 rpm of agitation. Subsequently, each strain was subcultured 1:1000 into fresh LB for a final volume of 150 μL using 96‐well microplates, using three wells per strain as technical replicates. Microplates were then incubated on a Spectramax 190 spectrophotometer (Molecular Devices, San Jose, CA) at 37°C, and the optical density at 600 nm (OD_600 nm_) of each well was measured every 5 min for 24 h, with 10 s of agitation before each read (Figure 2). Next, for each strain, the OD_600nm_ readings for all technical replicates were averaged and the OD versus time growth curves were plotted on a semi‐logarithmic graph to manually identify the exponential phase of each culture and calculate the generation time (g) as g = ln 2/growth rate. The growth rate (μ) was calculated as *μ* = ln (OD_600nm_ at the end of exponential phase/OD_600nm_ at the beginning of exponential phase)/(time at the end of exponential phase – time at the beginning of exponential phase). Final generation times were calculated by averaging the results from a total of four biological replicates, each with three technical replicates as described above.

### 2.4 Growth Curve Experiments Under Stress Conditions

These experiments were performed as described in the previous section, using four biological and three technical replicates, with the following modifications.

#### 2.4.1 Temperature Stress

After subculture, strains were incubated in the Spectramax 190 spectrophotometer set at 25, 30, or 42°C for 12–36 h with all other settings remaining the same.

#### 2.4.2 pH Stress

Strains were grown overnight in LB at 37°C and subcultured 1:1000 in LB adjusted to pH 6 or pH 9 using 1 N solutions of hydrochloric acid (HCl) or sodium hydroxide (NaOH), respectively. Additional pH‐stress growth experiments were conducted using buffered LB containing 150 mM MES (2‐(N‐morpholino)ehtanesulfonic acid) and adjusted to pH 6; and buffered LB containing 150 mM TRIS (tris(hydroxymethyl)aminomethane) adjusted to pH 7.4 (pH of regular LB) or pH 8.25. A pH of 8.25 instead of pH 9 was used for this experiment because no growth was observed in LB‐TRIS buffered to pH 9, 8.75, or 8.5. pH was measured using an Accumet Basic AB15 pH meter (Fisher Scientific, Waltham, MA). Growth in buffered LB at different pHs was assayed as a result of an additional experiment showing that growth of the parental strain in regular LB significantly impacted pH. LB has a limited buffering capacity and thus, as bacteria use nutrients and then consume acidic byproducts, its pH changes (Ratzke and Gore 2018). This change was measured by subculturing the parental strain grown in LB 1:1000 into a flask containing 500 mL of LB at 37°C. The culture was incubated with constant agitation (200 rpm) for 10 h. A 5 mL sample was taken aseptically from the flask every 20 min. From this sample, 150 µL were added to a single well of a sterile, 96‐well plate and the OD_600nm_ was read in the Spectramax spectrophotometer. The remainder of the sample was used to measure the pH using an Accumet Basic AB15 pH meter.

#### 2.4.3 Limited Oxygen Stress

Strains were prepared as described in Section 4.3 except for adding 100 µL of filter‐sterilized mineral oil on top of the 150 μl of each subculture microplate well to reduce oxygen diffusion during incubation in the Spectramax spectrophotometer.

#### 2.4.4 Nutritional Stress

Strains were grown as described in Section 4.3 and subcultured 1:1000 in BD Difco (Franklin Lakes, NJ, USA) M9 Minimal salts medium with 0.2% glucose or glycerol as the carbon and energy source, followed by incubation in the Spectramax spectrophotometer. M9 medium was prepared fresh by adding 4 mL Difco M9 Minimal salts 5x solution (33.9 g anhydrous disodium phosphate, 15.0 g monopotassium phosphate, 2.5 g sodium chloride, 5.0 g ammonium chloride per Liter), 40 µL of 1 M magnesium sulfate, 200 µL of 20% (w/v) filter‐sterilized glucose or glycerol (v/v), and sterile deionized water to 20 mL.

### 2.5 Statistical Analysis

Statistically significant differences between the parental strain and efflux mutants in motility or growth curve experiments were determined by *t*‐test (two independent samples with equal variance, two‐tailed distribution) using Microsoft® Excel 2021 software.

## 3 Results and Discussion

### 3.1 Swimming Motility Increases in Most TolC‐Dependent MDR Pump Mutants

**Figure 3:**
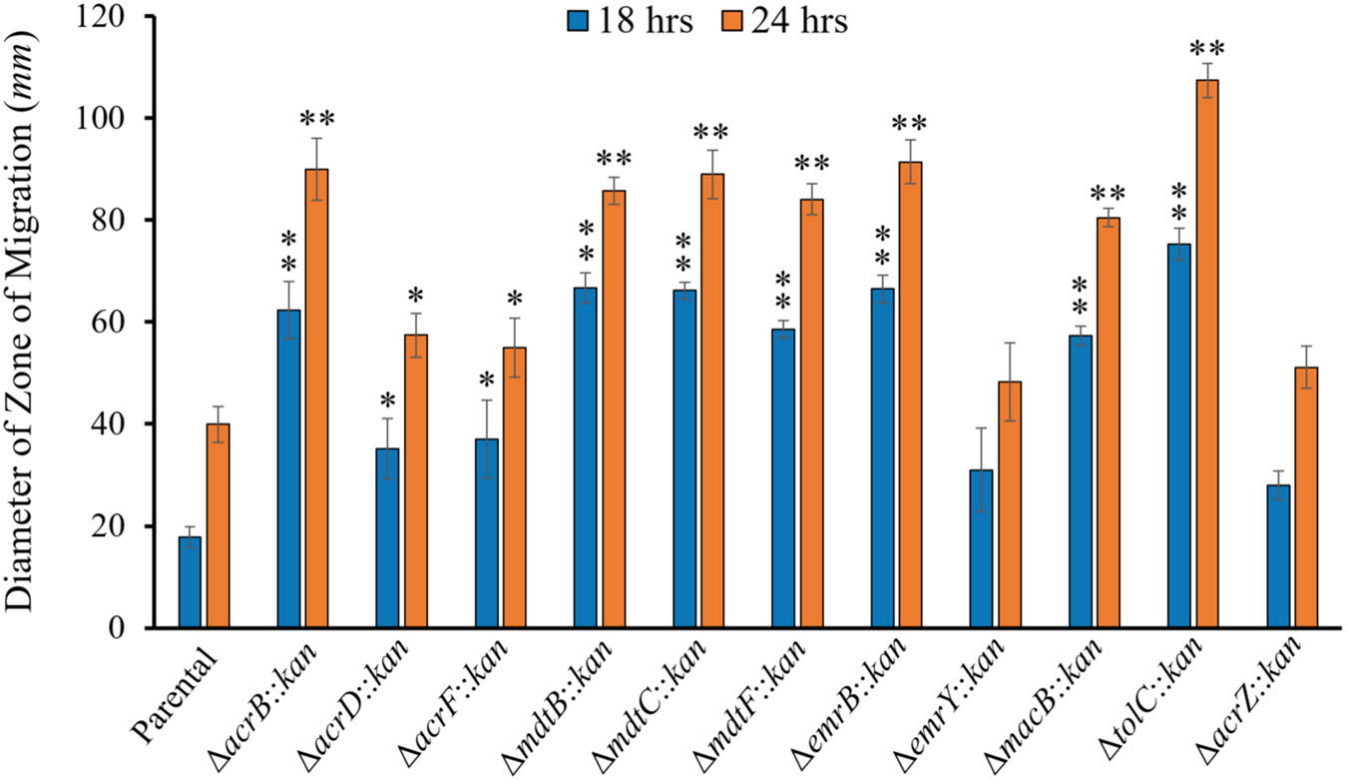
Swimming motility in TolC‐dependent multidrug efflux pump mutants increases for all mutants compared to the parental strain except for the Δ*emrY* and Δ*acrZ* mutants. The data are presented as average ± SEM of the diameter of the zone of migration in mm of each strain after 18‐ and 24 h of incubation. Results are from at least four biological replicates each with two technical replicates. Statistically significant differences between each mutant compared to the parental at the same time point are indicated as ** (*p* < 0.001) or * (*p* < 0.05).

*E. coli* mutants for the inner membrane transporter component of all eight TolC‐dependent MDR pumps, plus Δ*acrZ* and Δ*tolC* mutants were inoculated onto 0.3% agar plates and the diameter of the zone of motility was measured at 18‐ and 24‐h of incubation. Swimming motility significantly increased during both time measurements for all mutants tested compared to the parental strain except for the Δ*emrY* and Δ*acrZ* mutants (Figure 3). The results for the Δ*acrB* mutant are consistent with the increased expression of flagellum biosynthesis and motility genes, and the increased motility, we have previously described in the Δ*acrB* mutant (Ruiz and Levy 2014; Maldonado et al. 2023), as well as with the overexpression of flagellum and chemotaxis genes found in the all‐efflux pump‐knockout EKO‐35 mutant (Teelucksingh et al. 2022). However, increases in motility in the individual *E. coli* pump‐deletion mutants Δ*acrD*, Δ*acrF*, Δ*mdtB*, Δ*mdtC*, Δ*mdtF*, Δ*mdtB*, Δ*emrB*, and Δ*macB*, or the Δ*tolC* mutant, are described here for the first time. Interestingly, the Δ*macAB* mutant in *Serratia marcescens* has been shown to decrease swimming motility (Shirshikova et al. 2021), which suggests that the direction of the connection between MDR efflux pumps and motility is species dependent. Several factors may contribute to the increase in motility found here for most efflux mutants, as discussed below.

First, secondary mutations might have contributed to the increased motility found in one or more the efflux mutants tested. E.g. during long‐term adaptation experiments to cefotaxime in *Salmonella*, mutations in *envZ* that caused a downregulation of curli (an effect that is associated with increased motility) were found alongside *acrB* mutations in some lineages (Trampari et al. 2022).

Second, flagella and all the TolC‐dependent pumps except MacAB‐TolC are powered by the PMF (Du et al. 2018; Li, Plésiat, and Nikaido 2015). PMF consumption by AcrAB‐TolC, MdtEF‐TolC and EmrAB‐TolC has been hypothesized to impact their fitness contributions (Schaffner et al. 2021), and might also affect the function of flagella considering that the flagellar motor speed changes with the PMF (Gabel and Berg 2003). Therefore, it is possible that an increase in PMF available for flagella rotation contributes to the increase in swimming motility found here in most TolC‐dependent efflux mutants. In agreement with this hypothesis, a recent study has shown increased membrane potential in a *Salmonella* Δ*acrB* mutant (Whittle et al. 2024). However, this hypothesis would not directly explain the increase in motility found for the Δ*macB* and Δ*tolC* mutants. The energy source for the MacAB‐TolC pump is ATP‐hydrolysis and not PMF (Du et al. 2018; Li, Plésiat, and Nikaido 2015), although it is possible that less ATP consumption in the Δ*macB* mutant decreases PMF consumption used for ATP synthesis, and thus indirectly increases the available PMF. More importantly, in LB, deletion of *tolC* was found to not significantly increase the PMF or flagellar motor speed compared to the wild type (Le et al. 2021), which seems to discard the PMF hypothesis at least in the Δ*tolC* mutant.

Third, we have recently found that the *acrAB* transcriptional repressor AcrR is a direct repressor of the master regulator of flagellum biosynthesis and motility *flhDC* operon (Maldonado et al. 2023). Based on this finding, we proposed a model in which cellular metabolites that accumulate in the Δ*acrB* mutant act as ligands that bind to and inactivate AcrR, derepressing *flhDC* expression and thus increasing motility (Maldonado et al. 2023). In this model, insufficient efflux of metabolites that are toxic and/or whose accumulation disrupts metabolic flow would inactivate AcrR, causing an upregulation of efflux and motility genes to restore homeostasis and facilitate escape to a different environment (Maldonado et al. 2023). Consistent with this model, we have previously found that: 1) gene expression changes in the Δ*acrB* mutant were in part mediated by changes in the activity, not the expression, of AcrR (Ruiz and Levy 2014); 2) many cellular metabolites accumulate in the Δ*acrB* mutant (Cauilan et al. 2019); 3) metabolites such as malonate can inhibit the AcrAB‐TolC pump (Cauilan and Ruiz 2022); and 4) three cellular metabolites (polyamines) bind to and inactivate AcrR (Harmon and Ruiz 2022). Furthermore, we have also found that overexpressing *acrR* from a plasmid reduces motility in the Δ*acrB* mutant down to parental levels; and that the addition of the AcrR ligand ethidium bromide prevents such effect by AcrR in the Δ*acrB* mutant (Maldonado et al. 2023). We speculate that a similar model involving AcrR and/or other efflux transcriptional regulators such as EmrR may explain the increased motility found for the other TolC‐dependent pump mutants tested. EmrR, a transcriptional repressor of *emrAB* (Lomovskaya, Lewis, and Matin 1995), has been shown to regulate motility through the repression of *flhD* and *fliC* (Kakkanat et al. 2017).

Overall, it is also possible that the increased motility found in TolC‐dependent efflux mutants is the result of a combination of changes in PMF; secondary mutations; increased transcription of motility genes caused by the inactivation of regulators such as AcrR and EmrR by cellular metabolites that accumulate efflux mutants; and/or is caused by envelope stress responses such as the activation of the BaeSR and CpxARP regulatory systems previously found in Δ*tolC* mutants (Rosner and Martin 2013).

Interestingly, the Δ*emrY* and Δ*acrZ* mutants were the only ones that had motility similar to that of the parental strain (Figure 3). The small protein AcrZ does not directly bind to AcrAB‐TolC substrates but rather modulates AcrB binding to a subset of its substrates (Hobbs et al. 2012). Regarding EmrKY‐TolC, there is a high level of overlap in the efflux profiles of the TolC‐dependent MDR pumps (Li, Plésiat, and Nikaido 2015; Anes et al. 2015; Teelucksingh et al. 2022; Fernando and Kumar 2013). Additionally, AcrAB‐TolC is considered to be the primary pump for efflux in *E. coli* under laboratory conditions (Li, Plésiat, and Nikaido 2015; Anes et al. 2015; Tal and Schuldiner 2009), whereas EmrKY‐TolC did not affect the antibiotic susceptibility of *E. coli* in these conditions unless overexpressed in an AcrAB‐TolC‐free background (Nishino and Yamaguchi 2001). Moreover, expression levels of *emrKY* are low in laboratory conditions, only increasing within the macrophage or when exposed to acidic conditions (Pasqua et al. 2019; Kato et al. 2000). Thus, we hypothesize that, under the conditions tested, deletion of *emrY* or *acrZ* does not contribute enough to changes in PMF, intracellular metabolites, and/or envelop stress to trigger the aforementioned mechanisms suggested to cause increased motility in the other TolC‐dependent efflux mutants tested.

### 3.2 Growth of TolC‐Dependent MDR Pump Mutants in LB at 37°C Is Similar to That of the Parental Strain Unless All TolC‐Dependent Pumps Are Inactivated Simultaneously

**Figure 4:**
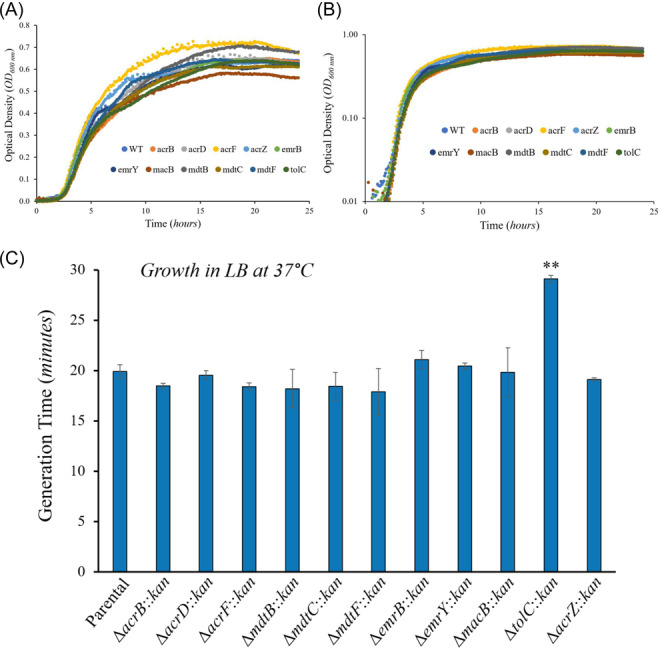
Growth in LB at 37°C decreases (longer generation time) in the Δ*tolC* mutant but not in the other TolC‐dependent multidrug efflux pump mutants tested compared to the parental strain. (A–C) Growth was measured as the optical density at 600 nm (OD_600nm_) every 5 min for 24 h. (A, B) Growth curves are shown with the Y axis (OD_600nm_) in both linear (A) and logarithmic (B) scales. For clarity reasons, only one biological replicate per strain, each including three technical replicates, is shown. (C) Generation time of the TolC‐dependent multidrug efflux pump mutants tested compared to the parental strain. Results are presented as the average generation time in minutes ± the SEM for each strain calculated using at least three biological replicate growth curves, each with three technical replicates. The generation times obtained were: 19.9 ± 0.7 (parental), 18.5 ± 0.2 (Δ*acrB*), 19.5 ± 0.4 (Δ*acrD*), 18.4 ± 0.4 (Δ*acrF*), 18.2 ± 1.9 (Δ*mdtB*), 18.4 ± 1.4 (Δ*mdtC*), 17.9 ± 2.3 (Δ*mdtF*), 21.1 ± 0.9 (Δ*emrB*), 20.5 ± 0.3 (Δ*emrY*), 19.8 ± 2.4 (Δ*macB*), 29.1 ± 0.3 (Δ*tolC*), and 19.1 ± 0.2 (Δ*acrZ*). Statistically significant differences between the generation time of the efflux mutants tested compared to the parental are indicated as ** (*p* < 0.01) or * (*p* < 0.05).

We next performed growth curve experiments in LB at 37°C to determine whether the increases in motility observed in Figure 3 for most efflux mutants were the result of faster growth of these mutants, as well as to investigate the contribution of each TolC‐dependent efflux pump to physiology and growth in *E. coli* under standard laboratory conditions (Figure 4). Interestingly, we found that the growth curves and generation times obtained for the parental strain, all individual TolC‐dependent MDR efflux pumps mutants, and the Δ*acrZ* mutant were very similar (Figure 4A–C). These findings are in agreement with the significant overlap in the efflux profiles of the TolC‐dependent pumps of *E. coli* (Li, Plésiat, and Nikaido 2015; Anes et al. 2015; Teelucksingh et al. 2022; Fernando and Kumar 2013), and suggest that the inactivation of individual pumps can be compensated by increased expression (Ruiz and Levy 2014) or activity of the other TolC‐dependent pumps of *E. coli*. Such redundancy among these efflux systems is also consistent with prior findings in *Salmonella* showing that inactivation of one or more of its TolC‐dependent RND pumps leads to an overexpression of its other TolC‐dependent RND pumps to compensate for such inactivation (Blair et al. 2015). In contrast, the Δ*tolC* mutant grew significantly slower (longer generation time) than the parental strain (Figure 4C), which discards faster growth as being the reason for the increased swimming motility found in the Δ*tolC* mutant (Figure 3). Slower growth in the Δ*tolC* mutant is in agreement with prior findings (Teelucksingh et al. 2022; Dhamdhere and Zgurskaya 2010), and seems to be the result of metabolic stress caused by the broad accumulation of cellular metabolites normally effluxed by TolC‐dependent pumps, envelope stress and increases in cell size (Rosner and Martin 2009; Cauilan et al. 2019; Horiyama and Nishino 2014; Rosner and Martin 2013; Dhamdhere and Zgurskaya 2010).

### 3.3 Individual TolC‐Dependent MDR Pumps Are Dispensable for Growth Under Stress Conditions, Whereas Simultaneous Inactivation of All TolC‐Dependent MDR Pumps Results in Slower Growth Under Most Stress Conditions

**Figure 5:**
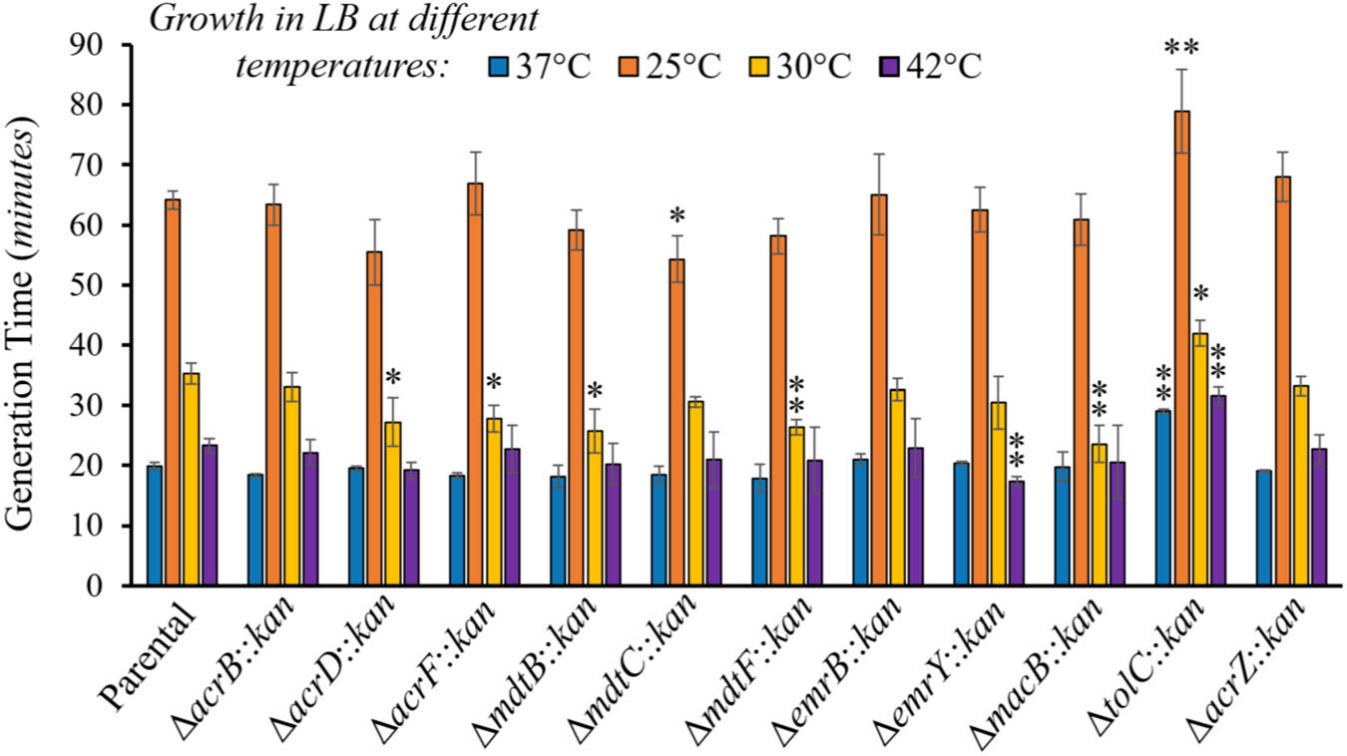
Growth of TolC‐dependent multidrug efflux pump mutants in LB at different temperature stresses. The growth of the Δ*tolC* mutant decreases (longer generation time) at both high and low‐temperature stress, whereas growth of the single‐pump mutants Δ*acrD*, Δ*acrF*, Δ*mdtB*, Δ*mdtF*, and Δ*macB* (at 30°C), Δ*mdtC* (at 25°C) and Δ*emrY* (at 42°C) increases (shorter generation times) compared to the parental strain. Growth was measured as the optical density at 600 nm (OD_600 nm_) every 5 min for 24 h. Results are presented as the average generation time in minutes ± the SEM for each strain calculated using at least four biological replicates, each including three technical replicates. The generation times obtained at 25°C were: 64.2 ± 1.5 (parental), 63.4 ± 3.3 (Δ*acrB*), 55.4 ± 5.4 (Δ*acrD*), 67.0 ± 5.2 (Δ*acrF*), 59.2 ± 3.3 (Δ*mdtB*), 54.3 ± 3.9 (Δ*mdtC*), 58.2 ± 2.9 (Δ*mdtF*), 65.1 ± 6.7 (Δ*emrB*), 62.5 ± 3.7 (Δ*emrY*), 60.8 ± 4.3 (Δ*macB*), 79.0 ± 6.9 (Δ*tolC*), and 68.0 ± 4.1 (Δ*acrZ*). The generation times obtained at 30°C were: 35.3 ± 1.7 (parental), 33.1 ± 2.4 (Δ*acrB*), 27.2 ± 4.0 (Δ*acrD*), 27.8 ± 2.3 (Δ*acrF*), 25.8 ± 3.6 (Δ*mdtB*), 30.6 ± 0.9 (Δ*mdtC*), 26.4 ± 1.3 (Δ*mdtF*), 32.6 ± 1.8 (Δ*emrB*), 30.5 ± 4.3 (Δ*emrY*), 23.6 ± 3.1 (Δ*macB*), 42.0 ± 2.2 (Δ*tolC*), and 33.3 ± 1.6 (Δ*acrZ*). The generation times obtained at 37°C are also shown for comparison and are reported in the legend of Figure 4. The generation times obtained at 42°C were: 23.3 ± 1.1 (parental), 22.2 ± 2.1 (Δ*acrB*), 19.3 ± 1.4 (Δ*acrD*), 22.8 ± 4.0 (Δ*acrF*), 20.3 ± 3.4 (Δ*mdtB*), 21.0 ± 4.6 (Δ*mdtC*), 20.9 ± 5.5 (Δ*mdtF*), 23.0 ± 4.9 (Δ*emrB*), 17.4 ± 0.7 (Δ*emrY*), 20.6 ± 6.1 (Δ*macB*), 31.6 ± 1.4 (Δ*tolC*), and 22.7 ± 2.5 (Δ*acrZ*). Statistically significant differences between the strains compared to the parental strain grown at the same temperature are indicated as ** (*p* < 0.01) or * (*p* < 0.05).

**Figure 6:**
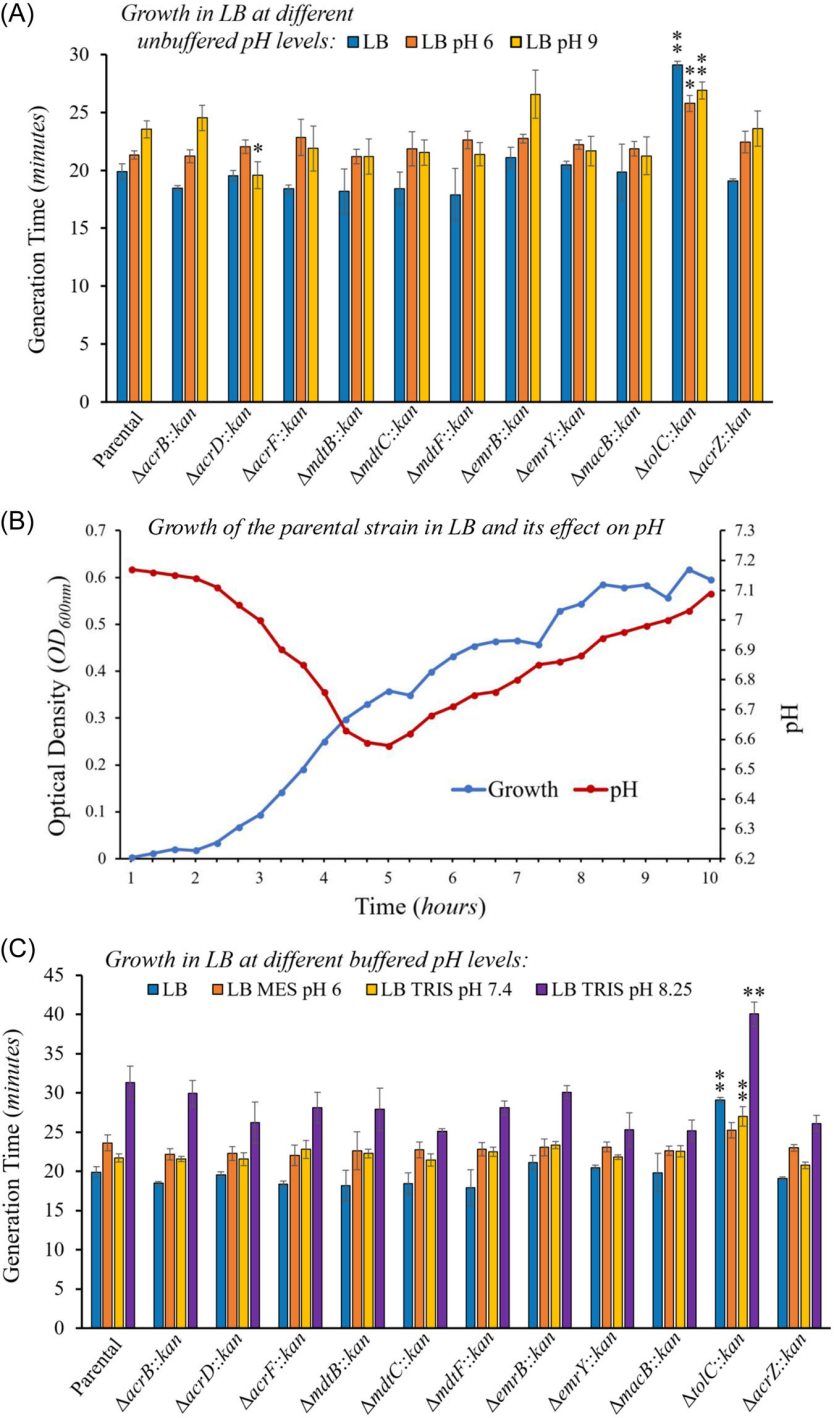
Growth of TolC‐dependent multidrug efflux pump mutants at 37°C in LB at different unbuffered and buffered medium pHs. The growth of the Δ*tolC* mutant decreased (longer generation time) under all pH conditions tested except for buffered pH 6, whereas the single pump‐deletion mutant Δ*acrD* was the only other mutant that grew faster (shorter generation time, and only at LB pH 9) compared to the parental strain. (A–C) Growth was measured as the optical density at 600 nm (OD_600 nm_) every 5 min for 24 h. Results are presented as the average generation time in minutes ± the SEM for each strain calculated using at least four biological replicates, each including three technical replicates. Statistically significant differences between the strains compared to the parental strain grown under the same pH conditions are indicated as ** (*p* < 0.01) or * (*p* < 0.05). (A) Unbuffered LB results. The generation times obtained in unadjusted LB (pH 7.4) are also shown for comparison and are reported in the legend of Figure 4. The generation times obtained in LB at pH 6 were: 21.3 ± 0.4 (parental), 21.2 ± 0.5 (Δ*acrB*), 22.0 ± 0.6 (Δ*acrD*), 22.8 ± 1.6 (Δ*acrF*), 21.2 ± 0.6 (Δ*mdtB*), 21.9 ± 1.5 (Δ*mdtC*), 22.6 ± 0.8 (Δ*mdtF*), 22.7 ± 0.4 (Δ*emrB*), 22.2 ± 0.4 (Δ*emrY*), 21.9 ± 0.6 (Δ*macB*), 25.8 ± 0.7 (Δ*tolC*), and 22.4 ± 0.9 (Δ*acrZ*). The generation times obtained in LB at pH 9 were: 23.5 ± 0.7 (parental), 24.5 ± 1.1 (Δ*acrB*), 19.6 ± 1.2 (Δ*acrD*), 21.9 ± 1.9 (Δ*acrF*), 21.2 ± 1.5 (Δ*mdtB*), 21.5 ± 1.1 (Δ*mdtC*), 21.4 ± 1.0 (Δ*mdtF*), 26.6 ± 2.1 (Δ*emrB*), 21.7 ± 1.3 (Δ*emrY*), 21.2 ± 1.6 (Δ*macB*), 26.9 ± 0.7 (Δ*tolC*), and 23.6 ± 1.5 (Δ*acrZ*). (B) Representative growth curve of the parental strain and its effect on the pH of regular LB medium. The medium pH decreases during the exponential phase and recovers during the stationary phase. Growth (optical density at 600 nm, OD_600nm_; left Y axis) and pH of the medium (right Y axis) were measured every 20 min for 10 h. (C) Buffered LB results. The generation times obtained in unbuffered LB (pH 7.4) are also shown for comparison The generation times obtained in LB‐MES buffered at pH 6 were: 23.6 ± 1.0 (parental), 22.1 ± 0.7 (Δ*acrB*), 22.3 ± 0.9 (Δ*acrD*), 22.0 ± 1.3 (Δ*acrF*), 22.6 ± 2.4 (Δ*mdtB*), 22.7 ± 1.0 (Δ*mdtC*), 22.8 ± 0.8 (Δ*mdtF*), 23.0 ± 1.1 (Δ*emrB*), 23.1 ± 0.6 (Δ*emrY*), 22.6 ± 0.6 (Δ*macB*), 25.2 ± 1.0 (Δ*tolC*), and 23.0 ± 0.4 (Δ*acrZ*). The generation times obtained in LB‐TRIS buffered at pH 7.4 were: 21.7 ± 0.5 (parental), 21.6 ± 0.3 (Δ*acrB*), 21.5 ± 0.8 (Δ*acrD*), 22.8 ± 1.1 (Δ*acrF*), 22.3 ± 0.6 (Δ*mdtB*), 21.4 ± 0.8 (Δ*mdtC*), 22.5 ± 0.6 (Δ*mdtF*), 23.4 ± 0.5 (Δ*emrB*), 21.8 ± 0.3 (Δ*emrY*), 22.5 ± 0.7 (Δ*macB*), 27.0 ± 1.2 (Δ*tolC*), and 20.8 ± 0.4 (Δ*acrZ*). The generation times obtained in LB‐TRIS buffered at pH 8.25 were: 31.3 ± 2.1 (parental), 29.9 ± 1.7 (Δ*acrB*), 26.2 ± 2.6 (Δ*acrD*), 28.1 ± 2.0 (Δ*acrF*), 27.9 ± 2.7 (Δ*mdtB*), 25.1 ± 0.3 (Δ*mdtC*), 28.1 ± 0.9 (Δ*mdtF*), 30.1 ± 0.8 (Δ*emrB*), 25.3 ± 2.1 (Δ*emrY*), 25.2 ± 1.3 (Δ*macB*), 40.1 ± 1.5 (Δ*tolC*), and 26.1 ± 1.0 (Δ*acrZ*).

**Figure 7:**
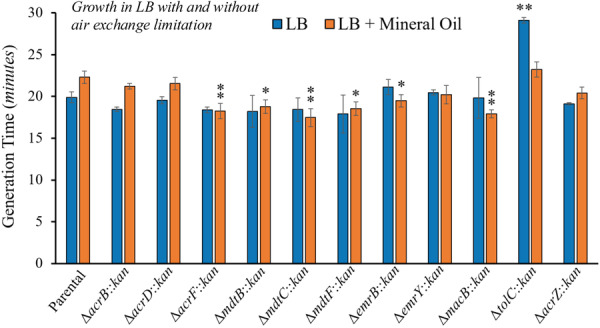
Growth of TolC‐dependent multidrug efflux pump mutants in LB at 37°C under limited air exchange (mineral oil covering) conditions. Growth in limited oxygen conditions restores growth of the Δ*tolC* mutant to the parental strain levels and increases growth (shorter generation) in the Δ*acrF*, Δ*mdtB*, Δ*mdtC*, Δ*mdtF*, Δ*emrB*, and Δ*macB* mutants. Growth was measured as the optical density at 600 nm (OD_600nm_) every 5 min for 24 h. Results are presented as the average generation time in minutes ± the SEM for each strain calculated using at least four biological replicates, each including three technical replicates. The generation times obtained in LB under aerobic conditions are shown for comparison and are reported in the legend of Figure 4. The generation times obtained under limited oxygen conditions were: 22.3 ± 0.7 (parental), 21.2 ± 0.3 (Δ*acrB*), 21.5 ± 0.7 (Δ*acrD*), 18.3 ± 0.9 (Δ*acrF*), 18.8 ± 0.8 (Δ*mdtB*), 17.5 ± 1.1 (Δ*mdtC*), 18.5 ± 0.8 (Δ*mdtF*), 19.5 ± 0.7 (Δ*emrB*), 20.2 ± 1.1 (Δ*emrY*), 17.9 ± 0.5 (Δ*macB*), 23.2 ± 0.9 (Δ*tolC*), and 20.4 ± 0.7 (Δ*acrZ*). Statistically significant differences between the strains compared to the parental strain grown under the same conditions are indicated as ** (*p* < 0.01) or * (*p* < 0.05).

**Figure 8:**
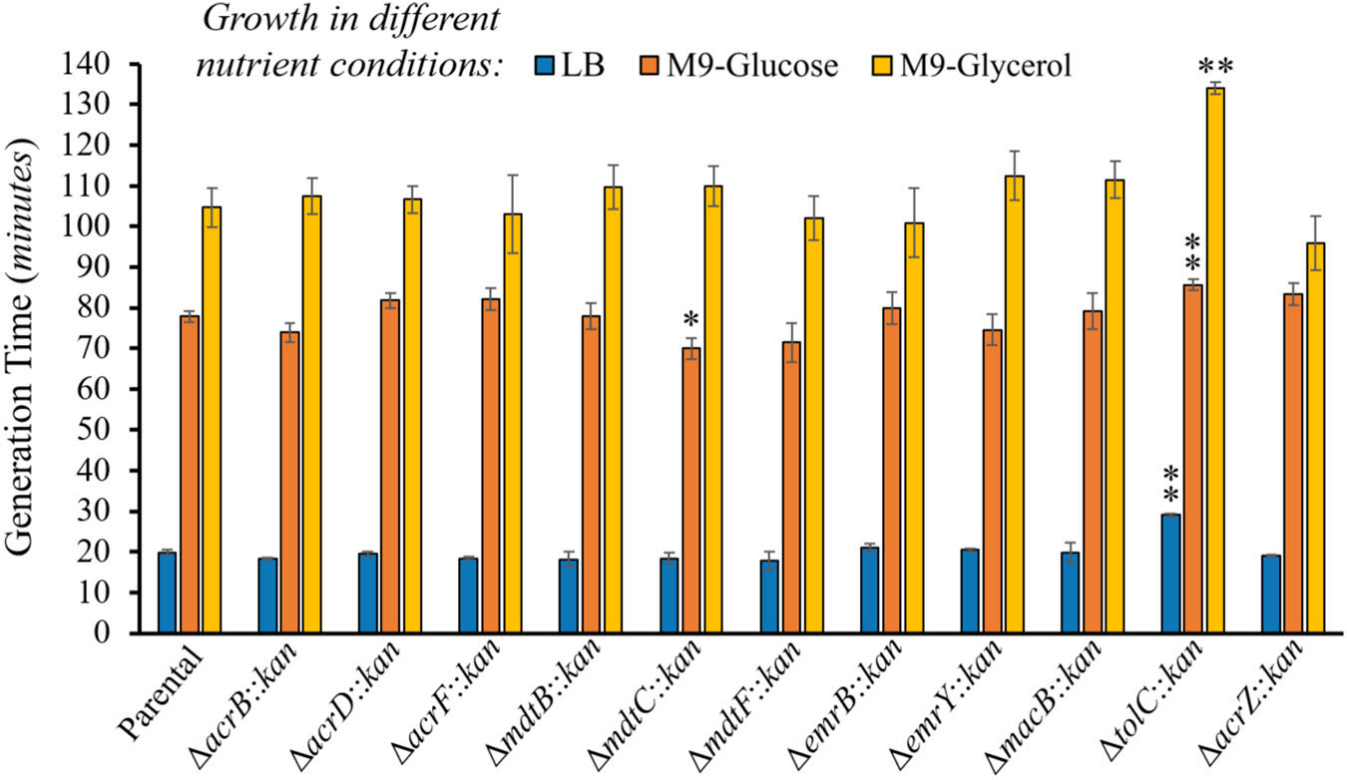
Growth of TolC‐dependent multidrug efflux pump mutants at 37°C under limited nutrient conditions. Growth in M9 minimal medium with 0.2% glucose or glycerol as the sole carbon and energy source was slower (longer generation time) in the Δ*tolC* compared to the parental, whereas only growth of the Δ*mdtC* mutant (shorter generation time in M9‐glucose) was affected for the other efflux mutants tested. Growth was measured as the optical density at 600 nm (OD_600nm_) every 5 min for 36 h. Results are presented as the average generation time in minutes ± the SEM for each strain calculated using at least four biological replicates, each including three technical replicates. The generation times obtained in LB under aerobic conditions are shown for comparison and are reported in the legend of Figure 4. The generation times obtained in M9‐glucose were: 77.8 ± 1.3 (parental), 73.9 ± 2.3 (Δ*acrB*), 81.7 ± 1.8 (Δ*acrD*), 82.1 ± 2.8 (Δ*acrF*), 77.8 ± 3.2 (Δ*mdtB*), 70.0 ± 2.6 (Δ*mdtC*), 71.4 ± 4.9 (Δ*mdtF*), 79.8 ± 4.0 (Δ*emrB*), 74.6 ± 6.1 (Δ*emrY*), 79.2 ± 4.4 (Δ*macB*), 85.6 ± 1.3 (Δ*tolC*), and 83.3 ± 2.7 (Δ*acrZ*). The generation times obtained in M9‐glycerol were: 104.7 ± 4.8 (parental), 107.4 ± 4.4 (Δ*acrB*), 106.6 ± 3.3 (Δ*acrD*), 103.1 ± 9.6 (Δ*acrF*), 109.8 ± 5.4 (Δ*mdtB*), 110.0 ± 4.9 (Δ*mdtC*), 102.0 ± 5.3 (Δ*mdtF*), 100.8 ± 8.5 (Δ*emrB*), 112.4 ± 6.1 (Δ*emrY*), 111.5 ± 4.5 (Δ*macB*), 134.1 ± 1.5 (Δ*tolC*), and 95.8 ± 6.6 (Δ*acrZ*). Statistically significant differences between the strains compared to the parental are indicated as ** (*p* < 0.01) or * (*p* < 0.05).

To further characterize the role of TolC‐dependent pumps under stress conditions, all efflux mutants were grown under various environmental stress conditions: temperature, pH, limited air exchange, and nutrient limitation (Figures 5, 6, 7, 8). Overall, individual pump mutants had no effect or in some cases had shorter generation times (faster growth) under several stress conditions, further supporting the hypothesis that changes in the expression and/or activity of these efflux pumps can compensate for each other because of their partially overlapping substrate profiles. In contrast, the Δ*tolC* mutant displayed longer generation times (slower growth; Figures 5, 6, 7, 8) than the parental in most stress conditions, suggesting that TolC‐dependent efflux is important for coping with environmental stress. Although beyond the scope of the current work, future work examining the contribution of these efflux systems to growth under the stress conditions tested here, using cultures that also contain bile salts that may impact membrane integrity and/or antibiotics, would further enrich our understanding of the physiological roles of these transporters. We speculate that, in the presence of bile salts or antibiotics, the effects on growth under the stress conditions tested here would likely be more pronounced for several of the TolC‐dependent efflux mutants studied here, especially the Δ*acrB* and Δ*tolC* mutants.

#### 3.3.1 Temperature Stress

Strains were first grown in LB at three additional different temperatures (25°C, 30°C, and 42°C; Figure 5). As expected, the 25°C and 30°C temperature conditions produced significantly slower generation times overall compared to growth at 37°C, while the growth of all strains at 42°C was similar to growth at 37°C (Figure 5). Compared to the parental strain, the generation time was significantly shorter for the Δ*mdtC* mutant at 25°C, the Δ*acrD*, Δ*acrF*, Δ*mdtB*, Δ*mdtF*, Δ*macB* mutants at 30°C, and the Δ*emrY* mutant at 42°C (Figure 5). In contrast, the Δ*tolC* mutant had longer generation times at all temperatures tested (Figure 5). The slower growth of the Δ*tolC* mutant at 25°C is in agreement with prior findings (Teelucksingh et al. 2022).

In general, faster growth of several individual TolC‐dependent efflux mutants under some temperature stress conditions (Figure 5) suggests that the loss of several of these proteins can confer a growth advantage as long as not all TolC‐dependent efflux is eliminated. MDR pumps require significant resources for their synthesis and function (Du et al. 2018; Li, Plésiat, and Nikaido 2015). We hypothesize that by no longer producing some of these MDR pumps, bacteria can devote more energy and resources to growth. Such fitness trade‐offs are in agreement with previous findings showing that the Δ*acrB* mutant outcompeted the wild‐type strain in the absence of antimicrobials (Langevin and Dunlop 2018). In addition, we also speculate that the lower metabolic rate of *E. coli* grown at lower temperatures might decrease the accumulation of metabolic intermediates or by‐products that are toxic or whose accumulation in efflux mutants would disrupt metabolic flow, thus decreasing the need for individual TolC‐dependent pumps. Moreover, in the gut, *E. coli* grows at 37°C and is exposed to antimicrobial compounds produced by the host such as bile salts. In contrast, lower temperatures mimic environments external to the host, where this constant antimicrobial pressure from bile salts is reduced, thus possibly decreasing the need for several TolC‐dependent pumps.

The Δ*tolC* mutant was the only strain to exhibit slower growth than the parental strain under all temperature conditions (Figure 5). These findings further support the hypothesis that TolC‐dependent efflux of metabolites that are toxic and/or whose accumulation disrupts metabolic flow is important for coping with environmental stress. In addition, other factors may contribute to the longer generation times found in this mutant. Deletion of *tolC* has been found to induce membrane and metabolic stress (Rosner and Martin 2013; Dhamdhere and Zgurskaya 2010; Zgurskaya et al. 2011), which may contribute to the slower growth found here for the Δ*tolC* mutant under all temperatures tested. Moreover, deletion of *tolC* under high‐temperature stress has been found to induce periplasmic stress and destabilize TolC‐dependent pump components that include AcrAB, MdtA, MdtEF, EmrA, and MacA (Mateus et al. 2018), which may also contribute to the slower growth found for the Δ*tolC* mutant at 42°C.

#### 3.3.2 pH Stress

We next studied the growth of TolC‐dependent efflux mutants under pH stress. In LB adjusted to either pH 6 or pH 9, only the Δ*acrD* mutant grew faster (at pH 9) than the parental strain, whereas the Δ*tolC* mutant had increased generation times (grew slower) than the parental at both pHs (Figure 6A). Interestingly, the Δ*tolC* mutant was also the only strain that grew faster at pH 6 and pH 9 than in unadjusted LB (pH 7.4). The interpretation of these results is complicated by the fact that as bacteria grow, they use nutrients and produce waste products that alter the pH of the medium (Ratzke and Gore 2018). An initial drop in the medium pH using *E. coli* grown in pH‐adjusted LB has also been previously established (Sanchez‐Clemente et al. 2020). To validate these prior findings, we grew the parental strain in unadjusted LB and measured the change in the medium pH during growth (Figure 6B). The transition from the lag phase to the exponential phase (2–2.5 h timepoint) initiated a sharp drop in pH as previously found (Sanchez‐Clemente et al. 2020). As growth continued, the initial pH dropped from around pH 7.2 to its lowest point of pH 6.6 at the transition from exponential to early stationary phase, followed by a pH increase during the stationary phase and reaching pH 7.1 after 10 h of growth (Figure 6B). These changes most likely occur because of the production of fermentation acid products during exponential growth, followed by uptake and metabolization of fermentation products and/or amino acid decarboxylation occurring during the stationary phase (Pinhal et al. 2019; Amarasingham and Davis 1965).

Therefore, to gain further insight, we repeated our growth experiments using buffered LB (Figure 6C). Interestingly, all strains except the Δ*tolC* mutant grew slightly slower in LB‐TRIS buffered to pH 7.4 than in regular LB pH 7.4, whereas no significant differences or slightly slower growth was generally found for all strains grown in LB‐MES pH 6 compared to unbuffered LB adjusted to pH 6 (Figure 6A–C). None of the strains tested, including the parental, were able to grow in LB‐TRIS buffered to pH 9 (or pH 8.75 or pH 8.5), perhaps because Tris buffer at basic pH has previously been shown to increase outer membrane permeability and the release of cell envelope components (Irvin, MacAlister, and Costerton 1981). Thus, growth in alkaline buffered stress was tested using LB‐TRIS pH 8.25. Even at this lower buffered pH of 8.25, growth for all strains was significantly slower than in unbuffered LB adjusted to pH 9 (Figure 6A–C).

Interestingly, in LB‐buffered conditions, the Δ*tolC* mutant still grew slower than the parental at all pHs tested, although the difference was small and not statistically significant in buffered LB at pH 6, whereas it was quite large in LB buffered to pH 8.25. In contrast, no other efflux mutant tested, including the Δ*acrD* mutant, showed growth that was significantly different from that of the parental strain at any of the pHs tested in buffered LB (Figure 6C). Overall, our findings for growth at different pHs in unbuffered and buffered LB indicate that, while individual TolC‐dependent efflux pumps are dispensable under mild acid or alkaline stress, TolC‐dependent efflux (and/or the other effects previously discussed for TolC) contribute to growth under mild acid, and especially under alkaline stress conditions. Our results for growth in mild acid are consistent with those by Deininger et al. (Deininger et al. 2011). showing that a Δ*tolC* mutant, but not the individual *acrB, acrD, emrB, emrY, macB, mdtC, mdtF*, or *acrF* mutants, had a growth defect at pH 5 in LBK medium (10 g/L tryptone, 5 g/L yeast extract, 7.45 g/L KCl). This study also reported that the Δ*tolC* mutant grew slower than the parental in LBK medium buffered at pH 4.5–6 (Deininger et al. 2011), in agreement with our findings at pH 6. In contrast, they found that the Δ*tolC* mutant did not grow slower than the parental in LBK buffered at pH 6.5–9 (Deininger et al. 2011), which differs from our findings in LB‐TRIS buffered at pH 8.25, perhaps because of the different growth medium and/or genetic background used. Furthermore, the authors showed that TolC is required for survival in extreme acid (pH 2.0) stress, and to a lesser degree, in pH 5.5. This role in survival at extreme acid pH was mediated by the EmrAB‐TolC and MdtABC‐TolC pumps, as well as by a requirement of TolC for induction of the expression of the GadAB glutamate decarboxylase acid‐resistance system (Deininger et al. 2011).

#### 3.3.3 Limited Air Exchange Stress

Considering that *E. coli* often grows in variable oxygen conditions both in the gut and outside the host (Singhal and Shah 2020), we next tested the effect on efflux mutants of growth with limited oxygen exchange by covering 96‐well cultures with a layer of sterile mineral oil. Mineral oil can be added to cultures to effectively diminish the diffusion rates between air and medium (Umehara and Aoyagi 2023). There have also been observed declines in respiratory rates of *E. coli* and diminished oxygen levels in LB throughout the progression of exponential phase (Riedel et al. 2013), which would be exacerbated under limited oxygen exchange conditions. Growth under this condition resulted in modestly slower growth (longer generation time) for the parental strain compared to growth in the absence of the mineral oil layer (Figure 7). This finding is consistent with the decrease in energy production expected as cells consume oxygen present in the LB medium during growth.

Of note, we also observed that the addition of the mineral oil layer resulted in shorter generation times compared to the parental strain in the Δ*acrF*, Δ*mdtB*, Δ*mdtC*, Δ*mdtF*, Δ*emrB*, and Δ*macB* mutants (Figure 7). As discussed in Section 3.3.1, faster growth of individual TolC‐dependent efflux mutants compared to the parental might be the result of decreased energy consumption in these mutants. Such a decrease in energy consumption would be particularly significant as oxygen levels and thus energy production decrease during growth with mineral oil. Interestingly, this was the only growth condition in which the growth of the Δ*tolC* mutant strain did not differ significantly from that of the parental. Furthermore, the generation time of this mutant was 6 min shorter when grown covered with mineral oil than without oil (Figure 7). These findings strongly support the important role of TolC‐dependent MDR pumps in mitigating oxidative stress, as has been previously suggested (Dhamdhere and Zgurskaya 2010; Zgurskaya et al. 2011). We hypothesize that lower aerobic metabolism and metabolic rates in cultures covered with mineral oil might decrease the accumulation of metabolic products that are toxic or disrupt the metabolic flow, especially reactive oxygen species and other byproducts of aerobic metabolism, thus decreasing the need for TolC‐dependent efflux.

#### 3.3.4 Limited Nutrient Stress

**Figure A1:**
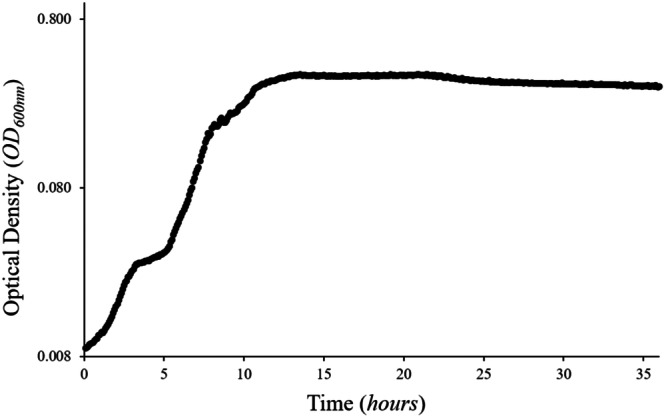
Representative diauxic growth curve for the Δ*tolC* mutant in M9 minimal medium with 0.2% glucose showing two defined lag and exponential phase stages before reaching sustained stationary phase. Growth was measured as the optical density at 600 nm (OD_600 nm_; Y axis, log scale) every 5 min for 36 h.

Finally, we tested the growth of TolC‐dependent efflux mutants in limited nutrient media. Growth curves in M9 minimal medium with glucose or glycerol as the sole carbon and energy source were diauxic for all strains tested, generally displaying two consecutive lag and exponential phases (Appendix Figure A1). Most likely, the first pattern of lag and exponential growth phases was produced as strains rapidly grew using glucose or glycerol. Once these initial carbon sources were mostly exhausted, bacteria would switch to growth on more partially oxidized byproducts of aerobic and fermentation metabolism, as previously suggested (Amarasingham and Davis 1965). As expected, all strains grew significantly slower (longer generation times) in M9‐glucose and M9‐glycerol than in LB (Figure 8; generation times shown are those obtained for the first exponential phase, when cells are primarily growing on glucose or glycerol).

Most efflux mutants had generation times very similar compared to the parental in both M9‐glucose and M9‐glycerol, except for the Δ*mdtC* mutant, which grew slightly faster in M9‐glucose, and the Δ*tolC* mutant, which grew slower in both M9‐glucose and M9‐glycerol (Figure 8). Our results with the Δ*tolC* mutant are in agreement with prior findings (Teelucksingh et al. 2022; Dhamdhere and Zgurskaya 2010; Zgurskaya et al. 2011). Overall, our findings again suggest that individual TolC‐dependent efflux pumps are dispensable under nutritional stress as long as not all of them are inactivated simultaneously, although other defects such as increased envelope and/or oxidative stress in the Δ*tolC* mutant may also contribute to the slower growth of this mutant, as we discussed in previous sections. Previous research has shown pleiotropic defects in Δ*tolC* mutants grown in M9‐glucose, including slower growth, abnormal cell division and morphology, inner membrane stress, and high NADH/NAD^+^ ratios caused by depletion of NAD^+^ resulting from inhibition of membrane NADH dehydrogenases upon transition to stationary phase (Dhamdhere and Zgurskaya 2010; Zgurskaya et al. 2011). A later study by Vega and Young (Vega and Young 2014) revealed that the addition of iron, or inactivation of enterobactin biosynthesis, restored the growth and cell morphology of the Δ*tolC* mutant in M9‐glucose to the parental levels. The authors suggested that the defects in this mutant in M9‐glucose were thus largely caused by the accumulation of enterobactin in the periplasm and subsequent sequestration of iron, potentially interfering with electron transport in the inner membrane and triggering the formation of reactive oxygen species (Vega and Young 2014). Considering that AcrAB‐TolC, AcrAD‐TolC and MdtABC‐TolC export enterobactin from the periplasm to the extracellular space (Horiyama and Nishino 2014), the findings of Vega and Young further support that lack of efflux is the primary driver for the reduced growth that occurs when the Δ*tolC* mutant is grown in M9‐glucose.

## 4 Conclusions

The eight TolC‐dependent multidrug efflux pumps of *E. coli* and other Enterobacteriaceae form a partially overlapping efflux system that plays a major role in resistance to antibiotics and bile salts. This system also impacts other functions such as metabolism, gene regulation, stress responses, and motility. However, the individual and combined physiological roles of these eight TolC‐dependent pumps under the same growth conditions and genetic background have not been fully characterized. This study helps fill this gap by examining the contribution to motility and growth under different stress conditions (temperature, pH, limited gas exchange, and limiting nutrients) of individual gene‐deletion mutants of the inner membrane transporter components for each TolC‐dependent pump, the small AcrB‐accessory protein AcrZ, and the TolC outer membrane channel. Our findings extend previous research and reveal novel insights into the physiological roles of these eight TolC‐dependent MDR pumps.

First, we show that the increase in swimming motility previously observed in Δ*acrB E. coli* mutants is AcrZ‐independent. Moreover, we report that individual inactivation of each TolC‐dependent pump except EmrKY‐TolC, or simultaneous inactivation of all of them using a Δ*tolC* mutant, increases swimming motility in *E. coli*. Current evidence suggests that this increase is mediated by regulators such as AcrR and EmrR, which directly repress both efflux and motility genes, likely in response to insufficient efflux. An increase in available PMF or other stress responses may also contribute to the observed motility changes.

Finally, we report that individual TolC‐dependent pumps are dispensable for growth under all stress conditions tested, whereas simultaneous inactivation of all eight pumps by deleting *tolC* slows down growth under all conditions tested except for limited air exchange. These findings support the hypothesis that due to their partially overlapping substrate profiles, changes in the activity and/or expression of these pumps can compensate for the inactivation of any individual pump. These changes, along with other motility, gene expression, and metabolic compensatory responses in individual efflux mutants, seem to maintain and, in some cases, even modestly increase the growth rate of *E. coli* under the different conditions studied, as long as not all TolC‐dependent pumps are simultaneously inactivated. However, additional factors such as envelope stress caused by the lack of TolC in the outer membrane may also contribute to some of the growth defects observed in the Δ*tolC* mutant. Future studies of these mutants under conditions that combine different environmental stresses, or under these stress conditions plus the presence of bile salts and/or other hazards, will further deepen our understanding of the individual and combined physiological roles of the eight TolC‐dependent MDR pumps of *E. coli*.

## Author Contributions

**Amanda M. Di Maso:** conceptualization, investigation, writing–original draft preparation. **Cristian Ruiz:** conceptualization, supervision, funding acquisition, writing–original draft preparation, writing–review & editing.

## Ethics Statement

The authors have nothing to report.

## Conflicts of Interest

The authors declare no conflicts of interest.

## Data Availability

All data are included within the article and its Appendix.
